# Effect of novel technology-enabled multidimensional physical activity feedback in primary care patients at risk of chronic disease – the MIPACT study: a randomised controlled trial

**DOI:** 10.1186/s12966-020-00998-5

**Published:** 2020-08-08

**Authors:** Oliver J. Peacock, Max J. Western, Alan M. Batterham, Enhad A. Chowdhury, Afroditi Stathi, Martyn Standage, Alan Tapp, Paul Bennett, Dylan Thompson

**Affiliations:** 1 Department for Health, University of Bath, Bath, BA2 7AY UK; 2 School of Health and Life Sciences, Teesside University, Middlesbrough, UK; 3 School of Sport, Exercise and Rehabilitation Sciences, University of Birmingham, Birmingham, UK; 4 Bristol Business School, University of West of England, Bristol, UK

**Keywords:** Physical activity, Technology, Primary care, Cardiovascular disease, Diabetes, Lifestyle intervention

## Abstract

**Background:**

Technological progress has enabled the provision of personalised feedback across multiple dimensions of physical activity that are important for health. Whether this multidimensional approach supports physical activity behaviour change has not yet been examined. Our objective was to examine the effectiveness of a novel digital system and app that provided multidimensional physical activity feedback combined with health trainer support in primary care patients identified as at risk of chronic disease.

**Methods:**

MIPACT was a parallel-group, randomised controlled trial that recruited patients at medium (≥10 and < 20%) or high (≥20%) risk of cardiovascular disease and/or type II diabetes from six primary care practices in the United Kingdom. Intervention group participants (*n* = 120) received personal multidimensional physical activity feedback using a customised digital system and web-app for 3 months plus five health trainer-led sessions. All participants received standardised information regarding physical activity. Control group participants (*n* = 84) received no further intervention. The primary outcome was device-based assessment of physical activity at 12 months.

**Results:**

Mean intervention effects were: moderate-vigorous physical activity: -1.1 (95% CI, − 17.9 to 15.7) min/day; moderate-vigorous physical activity in ≥10-min bouts: 0.2 (− 14.2 to 14.6) min/day; Physical Activity Level (PAL): 0.00 (− 0.036 to 0.054); vigorous physical activity: 1.8 (− 0.8 to 4.2) min/day; and sedentary time: 10 (− 19.3 to 39.3) min/day. For all of these outcomes, the results showed that the groups were practically equivalent and statistically ruled out meaningful positive or negative effects (>minimum clinically important difference, MCID). However, there was profound physical activity multidimensionality, and only a small proportion (5%) of patients had consistently low physical activity across all dimensions.

**Conclusion:**

In patients at risk of cardiovascular disease and/or type II diabetes, MIPACT did not increase mean physical activity. Using a sophisticated multidimensional digital approach revealed enormous heterogeneity in baseline physical activity in primary care patients, and practitioners may need to screen for low physical activity across dimensions rather than rely on disease-risk algorithms that are heavily influenced by age.

**Trial registration:**

This trial is registered with the ISRCTN registry (ISRCTN18008011; registration date 31 July 2013).

## Introduction

Low physical activity is a major public health problem and an important independent risk factor for chronic diseases such as cardiovascular disease (CVD) and type II diabetes [1]. Primary care provides a potential route to change physical activity in patients at risk of chronic disease. In the United Kingdom, National Health Service (NHS) Health Checks screen for at-risk adults and include brief advice on increasing physical activity [2]. However, past attempts to change physical activity in primary care have had only limited success [3]. Identifying strategies to increase physical activity in high-risk individuals in primary care settings remains a priority.

New digital technologies have the potential to transform the way in which physical activity is integrated into healthcare and used to target individuals at risk of long-term conditions [4]. Technology-assisted approaches can support more sophisticated, flexible and personalised delivery models [4–6] and can integrate key behavioural strategies associated with greater intervention effectiveness for modifying physical activity, including self-monitoring and feedback on behaviour [7, 8]. Indeed, a recent systematic review and meta-analysis called for future interventions aiming to promote longer term physical activity to specifically consider using personalised feedback as a behaviour change technique in order to enhance intervention effects [9].

Emerging evidence suggests that the health benefits of physical activity can be achieved in a variety of ways, and multiple dimensions (aspects) of physical activity are important for health promotion and disease prevention [10, 11]. Our past research shows that many people misjudge their physical activity status because they do not have access to accurate personalised information [12, 13]. We developed technology to capture multiple dimensions of physical activity and thus improve the depth and quality of feedback provided to individuals [14]. In addition to offering a more complete and integrated view of personal physical activity, a multidimensional approach potentially offers more behavioural options that can be tailored to an individual’s needs and preferences [10].

In the Multidimensional Individualised Physical ACTivity (MIPACT) study, we conducted a randomised controlled trial to examine whether technology-enabled feedback about personalised multidimensional physical activity combined with support from health trainers increased physical activity in patients at risk of cardiovascular disease and/or type II diabetes, recruited from primary care. This trial was funded by the National Prevention Research Initiative (NPRI) – a partnership of research councils, government, and medical charities in the United Kingdom prioritising disease prevention. Prior qualitative feedback from at-risk patients was used to inform the design of physical activity visualisations, the development of an accompanying web-based app for communicating novel multidimensional physical activity feedback and the inclusion of health trainer support in the present trial [12, 13].

## Methods

### Study design

The MIPACT study was a parallel group, single blind, randomised clinical trial enrolling 204 patients identified as being at risk of cardiovascular disease and/or type II diabetes, and consisted of a 3-month intervention period with follow-up at 3 and 12 months (primary timepoint). Ethics approval was obtained from the National Health Service (NHS) South West Research Ethics Committee (13/SW/0179). All patients provided written informed consent. The trial was registered on the ISRCTN registry on 31 July 2013 (ISRCTN18008011). A full protocol detailing trial methods has been published previously [15]. Data were collected and reported according to CONSORT guidelines (Additional file 1).

### Participants

Patients were recruited from six general medical practices in the South West of the United Kingdom between May 2014 and June 2015. Potential participants were approached via a letter from their General Practitioner. Eligible patients were men and women aged 40–70 years treated in primary care and with medium (≥10 and < 20%) or high (≥20%) risk of cardiovascular disease and/or type II diabetes. Risk scores were based on QRISK and QDiabetes prediction algorithms [16, 17] using clinical data. As our focus was on prevention, patients with existing coronary heart disease, heart failure, peripheral arterial disease, stroke, chronic kidney disease and diabetes mellitus were excluded. Individuals unable to increase physical activity or highly physically active individuals (a PAL – the ratio of total energy expenditure to basal metabolic rate – greater than 2.0) were also excluded.

### Randomisation and allocation

Eligible patients were allocated to one of two groups using an unequal allocation ratio (intervention: control) of 2:1, primarily to increase our experience with and amount of information on the new intervention with respect to delivery, receipt, and enaction [18]. Participants were allocated remotely by the trial statistician via concealed minimisation [19], providing balance across the trial arms for sex (male/female), general practice (1–5), risk (both cardiovascular disease and type II diabetes; medium/high) and baseline PAL (< 1.75 or > 1.75). Individual patients were the unit of randomisation, but there was no threat of contamination within a practice given that the intervention was personalised. Researchers assessing the primary outcomes were blinded to the allocation of participants.

### Intervention

The MIPACT intervention was a complex ‘treatment package’ developed in line with Medical Research Council guidelines for the development of complex interventions [20], and has been described previously [15] (see Additional file 2 for TIDieR checklist for intervention description). In brief, intervention content was developed by the project team and was informed by prior research on multidimensional physical activity and feedback in at-risk patient populations in primary care [12, 14]. We showed that at-risk groups were confident in using the technology and found feedback to be understandable and motivating. However, consistent with other qualitative work in at-risk patients [21] it was identified that health trainer support would be useful for sustaining motivation and engagement.

Behaviour change techniques common with successful lifestyle interventions were targeted by both the web-based app and the trainers [7]. The most prominent behavioural strategies in the web-based app (corresponding to an established taxonomy) [22], included feedback on behaviour (2.2), self-monitoring of physical activity behaviour (2.3), setting and reviewing goals in the context of target dimensions (1.1 & 1.5) and visualising the discrepancy between one’s behaviour and the health target (1.6). Health trainers were able to tailor the content of their discussions but were encouraged to discuss action planning/implementation intentions of physical activity (1.4), the health consequences of physical activity (5.1), instruction on how to perform specific physical activities (4.1) and building self-belief (15.1 & 15.3).

Recent research has shown that the use of multiple co-acting behaviour change techniques are required to promote motivationally adaptive healthcare environments [23, 24]. Thus, and in addition to the use of behaviour change techniques, a number of theoretically-informed intervention components based on self-determination theory were applied [25]. Within self-determination theory, healthcare exchanges that are supportive of an individual’s basic psychological needs for autonomy (i.e., need to feel that one’s behaviours are self-endorsed and volitional), competence (i.e., the need to feel effective and experience mastery in one's actions) and relatedness (i.e., the need to feel connected and cared for) are held to facilitate greater autonomous motivation and subsequently improved performance and increased maintenance of a given behaviour [26, 27]. The MiPACT intervention sought to support autonomy through the provision of behavioural choice inherent in the multidimensional physical activity profiles and via the trainer discussions wherein the users’ perspective of their current behaviour was explored, the use of a meaningful rationale, and encouragement to explore new enjoyable activities. Competence was targeted via the clear, visual feedback and self-monitoring of behaviour, and through the trainer supporting individuals in overcoming barriers to change, setting realistic goals and action plans, and by means of the provision of constructive verbal feedback and encouragement when talking through the visual data. Trainers were also encouraged to show empathy, acknowledge participant feelings, and build a collaborative relationship to support the need for relatedness.

All participants (including the control group) attended an initial 20-min meeting with a health trainer and received standardised information (including print-based materials and links to internet-based resources) regarding type II diabetes and cardiovascular disease, the potential benefits of physical activity on ‘risk’ reduction, current physical activity guidelines, ideas about getting more physically active, and included signposting to local opportunities.

Content was consistent with other print and internet-based resources such as NHS Choices (www.nhs.uk/livewell). Hence, the trial assessed effectiveness over-and-above existing ‘usual care’ alternatives. Control group participants received no further study intervention.

The MIPACT intervention involved access to a sophisticated wearable activity monitor and a customised web-based app for 3 months. The physical activity monitor was a multisensor device that estimates energy expenditure (BodyMedia Core). Participants were encouraged to wear activity monitors as much as possible and to regularly access the platform during the intervention. Personalised physical activity feedback could be accessed by participants using the web-based app at any point over a given 24-h period. Participants were also offered a further four (20–30 min) in-person health trainer sessions at approximately 2, 4, 8 and 12 weeks. Health trainers included 5 registered (e.g. dieticians) and non-registered healthcare professionals (e.g. health trainers) recruited from the local community with experience as physical activity or lifestyle advisors, in order to make the study as pragmatic and generalisable as possible to routine healthcare practice. Health trainers were provided written materials and 2-days of intervention delivery training; focusing on using an autonomy supportive style [15].

We included a range of strategies outlined by the National Institute of Health Behavior Change Consortium to reinforce intervention fidelity [28]. We (1) developed and implemented standardised training of intervention providers (2) trained more MIPACT trainers than needed to protect against dropout or illness (3) recorded a selection of consultation meetings for a sample of sessions per intervention provider (4) implemented fidelity checklists, and (5) provided formative feedback to health trainers. Engagement of participants with the use of activity monitors and the web-based app was assessed by the number of monitor wear days across the intervention, the number of days that monitor data were uploaded to the web-based app, and the total number of trainer sessions attended.

Activity monitor data were used to generate personal visual feedback and to enable remote self-monitoring through the app. Multidimensional physical activity data were depicted in a simple wheel and bar chart format using traffic light colour-coding as an index of attainment [15]. Each participant’s profile captured five different dimensions: (1) total energy expenditure, (2) minutes engaged in moderate-to-vigorous physical activity (MVPA) (3) time engaged in MVPA in ≥10-min bouts (4) time engaged in vigorous physical activity in ≥10-min bouts, and (5) sedentary time as a proportion of the waking day. The app included feedback on time spent and energy expended at different physical activity intensities (sedentary, light, moderate, vigorous and very vigorous), expressed in metabolic equivalents (METs). In order to convert energy expenditure to METs, we used age-specific equations for Basal Energy Expenditure [29]. This was used to determine the amount of time engaged in sedentary behaviour (< 1.8 METs), light intensity physical activity (1.8–3 METs), MVPA (≥3 METs) and vigorous intensity physical activity (≥6 METs). These data were presented as 24-h line graphs and daily/weekly summary charts using a ‘heat’ colour palette [15]. The app also offered reviewing and planning components, and functionality for tagging different behaviours as part of the self-monitoring process [15].

The aim of the first health trainer session was to explain the multidimensional nature of physical activity and physical activity intensity thresholds, discuss which personal behaviours contributed to each dimension and discuss acceptable and available options to increase daily physical activity. Participants were encouraged to engage in new and enjoyable activities. A focus of the second session was to review progress and to discuss aspects that participants would consider changing and to set a SMART (Specific, Measurable, Attainable, Realistic, and Time-bound) goal. Subsequent sessions involved reviewing physical activity behaviour(s) and supporting efforts to be more active and included continued support in refining goals and action plans. The process was led by the participant (i.e. “self-regulated”) and was highly individualised.

### Outcome measures

All participants were assessed at baseline and followed up at 3 and 12 months within clinic. The primary outcome was device-based assessment of physical activity at 12 months. The device used to collect physical activity energy expenditure was a research-grade multisensor instrument worn on the upper arm (BodyMedia Core, BodyMedia Inc., Pittsburgh, PA) that has been used in research studies and has excellent reported accuracy [30, 31]. Underlying raw minute-by-minute estimates of energy expenditure were extracted in order to undertake the necessary data processing to then extract the specific physical activity metrics needed for our analysis (SenseWear® Pro 8.0, algorithm v5.2). Weekly physical activity energy expenditure records were exported to Excel for processing. Data were considered valid if the participant wore the device for ≥6 days in which 80% or more data were captured within a given 24-h period. Missing data were assigned estimated energy expenditure equivalent to basal metabolic rate (Schofield et al., 1985). Physical Activity Level (PAL) was determined as the product of Total Energy Expenditure/Basal Metabolic Rate. Multiple other key physical activity dimensions were calculated, including: overall physical activity energy expenditure (expressed as PAL); accumulated minutes engaged in MVPA (≥3 METs) and in ≥10-min bouts; time engaged in vigorous intensity physical activity (≥6 METs) in ≥10-min bouts, and sedentary time (< 1.8 METs), where one MET represents resting metabolic rate.

As per study protocol [15], to explore if any change in physical activity is meaningful in terms of health risk, we included secondary outcomes for the change between baseline and 12 months in body mass, body mass index, waist circumference, fat mass, systolic and diastolic blood pressure, glucose control (glucose and insulin), lipids (total cholesterol, high-density and low-density lipoprotein cholesterol, triglycerides), and C-reactive protein. Anthropometric measurements and blood samples were taken by a research nurse in clinic, while body composition was estimated using dual energy X-ray absorptiometry. Risk scores were based on QRISK and QDiabetes prediction algorithms [16, 17] using clinical data. Health status was assessed using the Euroqol 5-D visual analogue scale (EQ. 5-D) [32]. A full process evaluation for MIPACT, including self-reported psychosocial variables, will be published separately.

### Statistical analysis

For a targeted difference in PAL of 0.07 and an SD of 0.18, [15] 2-sided *P* = 0.05, 90% power, an assumed correlation between baseline and follow-up of r = 0.7, and a 2:1 allocation ratio, the required sample size (allowing for 25% attrition) was 144 in the intervention group and 72 in the control (ANCOVA model) [33]. In an as-randomised analysis, we estimated the mean difference in 12-month outcome between arms. We applied a linear mixed model with restricted maximum likelihood and an identity covariance structure. All minimization factors and the baseline value of the outcome were included as fixed effects, plus a random slope for group allowing different response variances. The same analysis was applied to all physical activity dimensions, with no adjustment for multiplicity [34].

Our definition of the minimum clinically important difference (MCID) for MVPA was based on a prospective study of adults at risk of type 2 diabetes mellitus, wherefrom we can derive that the mean increase in MVPA time per day associated with a clinical meaningful 10% relative risk reduction in all-cause mortality was approximately 20% [35]. In the current study, 20% of the baseline mean equated to around 30 min/day and 20 min/day, respectively, for MVPA and for MVPA in 10-min bouts. The MCID for vigorous physical activity in 10-min bouts was set at half of that for MVPA (10 min/day), as there is no robust clinical anchor. The MCID for PAL was defined as 0.07, as detailed in our protocol paper [15], and for sedentary time as 1 h per day, as there is no robust clinical anchor independent of MVPA.

Model specification was evaluated by visual inspection of residuals plots. Mean intervention effects are presented with 2-sided 95% confidence intervals, providing a range of effect sizes compatible with the data and model. A zero effect ± the MCID defines a region of practical equivalence. The disposition of the confidence interval to this region may be used to statistically rule out substantial beneficial (favouring intervention) or harmful (favouring control) effects, equivalent to two 1-sided tests each at the 0.025 level [36]. In short, if the entire confidence interval lies within the region of zero ± the MCID then the groups may be considered practically equivalent for that outcome. The same analyses were repeated for the 3-month physical activity outcomes and for the health-related outcomes at 3 and 12 months, and are presented for description only.

With missing data on the dependent variable only, maximum likelihood reduces to a complete case analysis under a plausible missing at random assumption, and therefore cases with missing outcome data (c. 10%) were deleted. Finally, in our trial protocol we stated that we would explore treatment effect heterogeneity. However, when the mean intervention effect is close to zero, the plausibility of the implicit assumption that a proportion of participants would get substantially worse with the intervention is questionable. Therefore, we omitted the planned analysis of intervention response heterogeneity. All analyses were performed using Stata software (StataCorp, 2017. Stata Statistical Software: Release 15, College Station, TX, USA: StataCorp LP).

### Patient and public involvement

The public were involved in this study in several ways. Draft graphics and visualisations for presenting multidimensional physical activity data (initially developed alongside designers) were refined based upon feedback from in-depth qualitative interviews in at-risk patients (*n* = 29) and healthcare practitioners (*n* = 15) from two regions in the United Kingdom (Bath and North East Somerset and Wiltshire). Overall, patients preferred simple messages rather than more complex or abstract visualisations. Also, while technology-enabled physical activity feedback was found to be informative, understandable and motivating, it was the view of patients and practitioners that supplementary in-person guidance may further support behaviour change and this informed the decision to include one-to-one health trainer sessions in MIPACT [12]. Participants were provided with their results and were invited to attend focus groups at the end of the study to learn more about their experiences of the trial.

## Results

### Participants

**Fig. 1:**
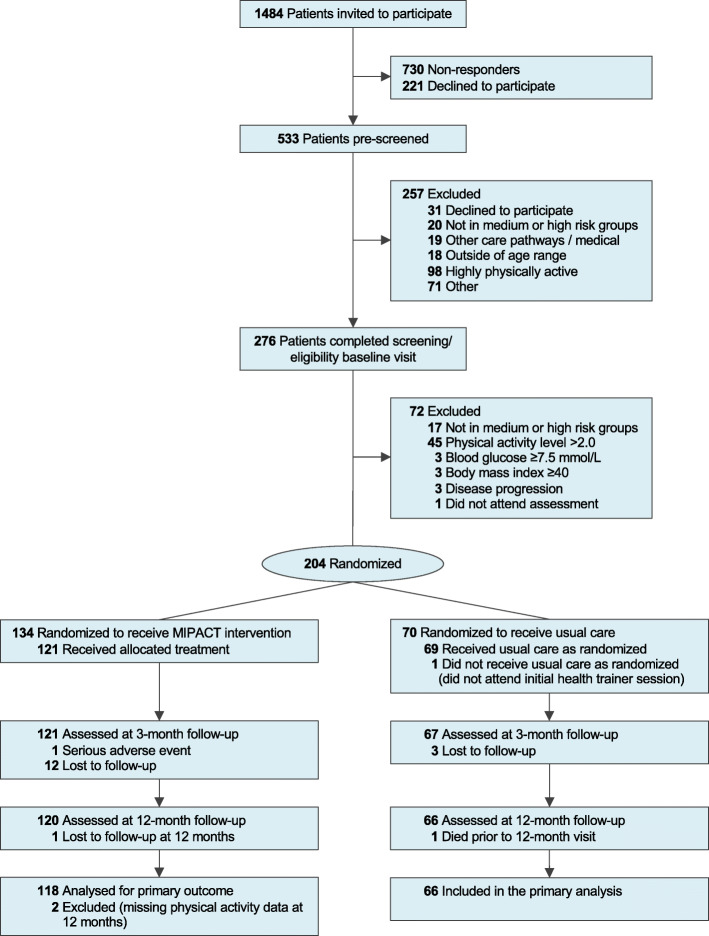
Flow of participants through the MIPACT study. MIPACT, Multidimensional Individualized Physical ACTivity

The flow of participants from recruitment through to final assessment at 12 months is shown in Fig. 1. Participant characteristics were similar between groups at baseline (Table 1).

**Table 1:** Characteristics of study participants^a, b^

Variable	Total (***n*** = 204)	Control (***n*** = 70)	Intervention (***n*** = 134)
**Age (years)**	64 (6)	63 (6)	64 (6)
**Male sex**	131 (64)	45 (64)	86 (64)
**Ethnicity:** White British	180 (88)	63 (90)	117 (87)
**Marital Status:** Married	150 (74)	54 (77)	96 (72)
**Employment status**			
In full or part-time employment	85 (42)	31 (44)	54 (40)
Retired	116 (57)	38 (54)	78 (58)
**Area deprivation (IMD score)**	7.6 (2.3)	7.7 (2.4)	7.6 (2.3)
**Education levels**			
Up to age 16 or less	63 (31)	18 (26)	45 (34)
Up to age 18	60 (29)	26 (37)	34 (25)
Undergraduate / higher degree	81 (40)	26 (37)	55 (41)
**Current smoker**	20 (10)	10 (14)	10 (7)
**Body mass (kg)**	85.2 (14.3)	86.6 (14.1)	84.5 (14.4)
**Body mass index (kg/m**^**2**^**)**	29.1 (4.4)	29.4 (4.3)	28.9 (4.4)
**DEXA**			
Total body fat (%)	33.4 (8.1)	33.0 (8.5)	33.5 (7.9)
Visceral body fat (cm^2^)	188 (60)	195 (66)	184 (57)
Fat mass index (kg/m^2^)	9.7 (3.5)	9.7 (3.6)	9.7 (3.4)
**Waist circumference (cm)**	100.3 (10.6)	101.2 (10.6)	99.8 (10.5)
**Blood pressure (mm Hg)**			
Systolic	132 (16)	132 (17)	132 (16)
Diastolic	84 (9)	83 (10)	84 (9)
**Fasting glucose (mmol/L)**	5.1 (0.5)	5.1 (0.5)	5.1 (0.4)
**Fasting insulin (mU/L) median (IQR)**	54.8 (41.1, 82.2)	58.9 (37.5, 84.1)	51.4 (42.4, 80.4)
**HOMA-IR median (IQR)**	1.8 (1.3, 2.7)	1.9 (1.2, 2.8))	1.7 (1.3, 2.6)
**Cholesterol (mmol/L)**			
Total cholesterol	5.8 (1.0)	5.7 (0.9)	5.9 (1.1)
Low-density lipoprotein cholesterol	3.6 (0.9)	3.5 (0.9)	3.7 (1.0)
High-density lipoprotein cholesterol	1.6 (0.4)	1.5 (0.4)	1.6 (0.5)
**Triglycerides (mmol/L)**	1.4 (0.6)	1.4 (0.7)	1.4 (0.6)
**QRISK2:** 10-year risk (%)	14.4 (6.3)	13.8 (6.2)	14.8 (6.4)
**QDiabetes:** 10-year risk (%)	13.5 (9.4)	12.9 (6.9)	13.9 (10.5)
**Physical Activity**			
Sedentary time (min/day)	662 (111)	651 (117)	668 (107)
MVPA (min/day)	149 (57)	157 (60)	146 (56)
MVPA10 (min/day)	96 (47)	103 (52)	92 (44)
Vigorous10 (min/day) median (IQR)	3 (0, 9)	3 (0, 11)	3 (0, 8)
PAL	1.68 (0.16)	1.70 (0.16)	1.67 (0.16)
**EQ 5-D VAS**	72 (16)	73 (16)	71 (15)

*Abbreviations*: *IMD* Index of multiple deprivation, *DEXA* Dual-energy X-ray absorptiometry, *HOMA-IR* Homeostatic model assessment – Insulin resistance, *PAL* Physical activity level, *EQ. 5-D VAS* Euroqol 5-D visual analogue scale (health-related quality of life)

^**a**^ Data are expressed as mean (SD) for continuous data, or N (%) for categorical data. Highly skewed continuous data are presented as median (interquartile range; IQR)

^**b**^*N* = 186 for DEXA; *N* = 204 for all other variables

### Effect of the intervention on physical activity outcomes

Adjusted means for device-measured physical activity at 12 months (primary endpoint) and 3 months are presented in Table 2. For the primary timepoint at 12 months, the point estimates and lower and upper confidence limits for the intervention effect (vs. control) for all dimensions were all trivial in relation to the smallest effect sizes of interest. For all physical activity dimensions the confidence interval is entirely within the equivalence region (defined by ± the MCID), indicating that clinically meaningful mean population effects (as defined) may be ruled out at the given level of error control.

**Table 2:** Adjusted means for device-based physical activity outcomes at 3 months and 12 months^a,b^ (All outcomes in minutes/day unless stated)

Variables	Intervention	Control	Difference (95% CI)
Sedentary			
3 months	651.1	632.5	18.6 (−8.9 to 46.2)
12 months	667.1	657.1	10.0 (−19.3 to 39.3)
MVPA			
3 months	163.4	174.8	−11.4 (−27.7 to 4.9)
12 months	161.9	163.0	−1.1 (− 17.9 to 15.7)
MVPA_10_			
3 months	112.4	115.1	−2.7 (−17.2 to 11.9)
12 months	107.0	106.8	0.2 (−14.2 to 14.6)
Vigorous_10_			
3 months	9.8	8.1	1.7 (−1.6 to 4.1)^c^
12 months	8.1	6.3	1.8 (−0.8 to 4.2)^c^
PAL			
3 months	1.72	1.74	−0.02 (− 0.07 to 0.03)
12 months	1.71	1.71	0.00 (−0.036 to 0.054)

*Abbreviations MVPA* moderate-to-vigorous physical activity, *MVPA*_*10*_ moderate-to-vigorous physical activity in bouts of at least 10 min, *Vigorous*_*10*_ Vigorous physical activity in bouts of at least 10 min, *PAL* Physical Activity Level (ratio of total daily energy expenditure to resting metabolic rate)

^a^ Adjusted for all minimization factors and baseline value of the outcome variable

^b^*N* = 183 for sedentary time at 3 months; *N* = 184 for all other variables at 3 and 12 months

^c^ Confidence interval verified using a bias-corrected and accelerated bootstrap with 2000 replication

### Effect of the intervention on health outcomes

Adjusted means for health-related outcomes are shown in Table 3. These data are provided for descriptive/exploratory purposes only.

**Table 3:** Adjusted means for health-related outcomes at 3 and 12-months^a^

Variable	N	Intervention	Control	Difference (90% CI)
Body mass (kg)				
3 months	188	84.1	84.3	−0.2 (−1.0 to 0.5)
12 months	186	83.6	84.4	−0.8 (−1.8 to 0.2)
Body mass index (kg/m^2^)				
3 months	188	28.6	28.7	−0.1 (− 0.3 to 0.1)
12 months	186	28.5	28.8	−0.3 (− 0.6 to 0.04)
Waist circumference (cm)				
3 months	188	98.3	98.0	0.3 (−0.6 to 1.2)
12 months	186	99.2	99.0	0.2 (−1.2 to 1.5)
Total body fat (%)				
3 months	165	32.6	32.5	0.1 (−0.4 to 0.6)
12 months	166	32.4	32.7	−0.3 (− 0.8 to 0.3)
Visceral body fat (cm^2^)				
3 months	165	180.4	183.1	−2.7 (− 8.8 to 3.4)
12 months	166	186.9	193.1	−6.2 (− 13.8 to 1.4)
Fat mass index (kg/m^2^)				
3 months	165	9.3	9.3	0.0 (−0.2 to 0.2)
12 months	166	9.3	9.5	−0.2 (− 0.4 to 0.1)
SBP (mmHg)				
3 months	188	129	130	−1 (−5 to 3)
12 months	186	133	133	0 (−3 to 3)
DBP (mmHg)				
3 months	188	83	85	−2 (−4 to 0)
12 months	186	82	83	−1 (−3 to 1)
Glucose (mmol/L)				
3 months	188	5.1	5.1	0.0 (−0.1 to 0.1)
12 months	183	5.1	5.2	−0.1 (− 0.2 to 0.0)
Insulin (mU/L)^b^				
3 months	183	65.8	68.3	−2.5 (−8.7 to 4.0)
12 months	181	72.2	71.7	0.5 (−7.9 to 9.6)
HOMA-IR^b^				
3 months	183	2.2	2.2	0.0 (−0.3 to 0.2)
12 months	177	2.4	2.4	0.0 (−0.4 to 0.3)
Total cholesterol (mmol/L)				
3 months	188	5.6	5.7	−0.1 (− 0.2 to 0.1)
12 months	184	5.7	5.8	−0.1 (− 0.3 to 0.05)
LDL cholesterol (mmol/L)				
3 months	188	3.4	3.4	0.0 (−0.2 to 0.2)
12 months	184	3.4	3.5	−0.1 (− 0.2 to 0.1)
HDL cholesterol (mmol/L)				
3 months	188	1.6	1.6	0.0 (−0.06 to 0.04)
12 months	184	1.6	1.6	0.0 (−0.1 to 0.03)
Triglycerides (mmol/L)^b^				
3 months	188	1.4	1.5	−0.1 (− 0.25 to 0.03)
12 months	184	1.4	1.5	−0.1 (− 0.2 to 0.1)
CRP (mg/l)^b^				
3 months	186	2.5	3.3	−0.8 (− 1.5 to − 0.1)
12 months	183	3.2	3.1	0.1 (−0.7 to 0.9)
CVD risk: 10-year risk (%)^b^				
3 months	188	14.0	14.0	0.0 (−0.5 to 0.5)
12 months	186	15.0	14.9	0.1 (−0.5 to 0.7)
Diabetes risk: 10-year risk (%)^b^				
3 months	188	12.8	13.0	−0.2 (− 0.7 to 0.3)
12 months	186	12.9	13.6	−0.7 (−1.5 to 0.02)
EQ-5D				
3 months	184	77.4	74.3	3.1 (0.0 to 6.2)
12 months	186	75.7	72.4	3.3 (−1.4 to 8.2)

*Abbreviations*: *SBP* Systolic blood pressure, *DBP* Diastolic blood pressures, *HOMA-IR* Homeostatic model assessment – Insulin resistance, *LDL* Low density lipoprotein, *HDL* High density lipoprotein, *CRP* C-reactive protein, *CVD* Cardiovascular disease, *EQ. 5D* Health related quality of life

^a^ Adjusted for all minimization factors and baseline value of the outcome variable

^b^ Confidence interval verified using a bias-corrected and accelerated bootstrap with 2000 replications

### Adherence and adverse events

The dose and coverage of intervention delivery was high. Among those receiving the MIPACT intervention, the mean (SD) number of days that activity monitors were worn across the 3-month intervention period was 72 (25) days. Of these, the number of days regarded as complete (i.e. ≥80% data for a given 24-h period) was 61 (26) days. On average, participants uploaded data to the platform on 24 (21) unique days. Of the 134 participants in the MIPACT intervention group, 121 (90%) attended ≥4 health trainer sessions, and 105 (78%) attended all 5 training sessions. After excluding participants who dropped out from the study, 85% attended all sessions. One participant in the control group died due to reasons deemed unrelated to the study. In total, there were only 10 serious adverse events reported between the intervention and control groups: 7 (5%) of 134 and 3 (4%) of 70, respectively, and none were deemed related to participation in the study.

## Discussion

In primary care patients at risk of chronic disease, we evaluated an intervention comprising personal technology-enabled feedback on multiple specific dimensions of physical activity combined with health trainer support. At 12-months follow-up, the intervention and control groups were statistically equivalent for all physical activity outcomes and sedentary time. Between participants there was considerable variation (or heterogeneity) across the multiple physical activity dimensions, with only 11 participants (5%) presenting at baseline with uniformly low physical activity across all dimensions.

### Comparison with other studies

We developed a highly sophisticated system for providing personalised multidimensional physical activity feedback that was informed by patients and practitioners (MIPACT). This approach did not change physical activity in at-risk patients recruited from primary care. MIPACT confirms that, even using the latest technology as part of a multicomponent intervention, it is hard to motivate people at risk of disease to change their physical activity behaviours. The MIPACT trial represents a first attempt to leverage technology to improve the depth and quality of feedback to at-risk patients about the multiple dimensions of physical activity that can play a role in disease prevention [10, 11, 14]. To date, physical activity feedback interventions have typically focused on just one aspect of physical activity behaviour (e.g. moderate physical activity). In a primary care setting, there is some evidence that personalised physical activity feedback on MVPA [37] or pedometer steps [38] as part of more intensive lifestyle interventions can lead to increases in physical activity. However, these effects were modest, and studies targeted patients with existing chronic disease. No prior randomised trial has examined the effects of providing personal feedback on multiple physical activity dimensions as part of a technology-enabled behavioural intervention in at-risk patient populations.

### Meaning of the study: possible explanations and implications

**Fig. 2:**
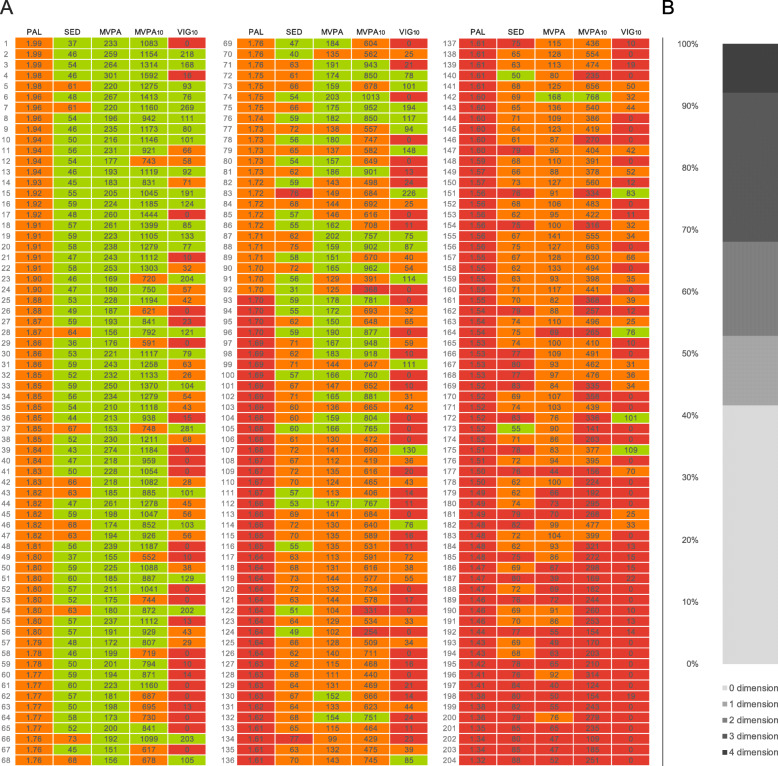
Multidimensional Physical Activity Profiles of Participants at Baseline. **a** Unadjusted physical activity data of participants across multiple specific dimensions of physical activity at baseline **b** Proportion of participants scoring highly for one or more dimensions of physical activity at baseline. PAL, Physical Activity Level (ratio of total daily energy expenditure to resting metabolic rate); SED, Sedentary; MVPA, moderate-to-vigorous physical activity; MVPA_10_, moderate-to-vigorous physical activity in bouts of at least 10 min; VIG_10_, Vigorous physical activity in bouts of at least 10 min. In this simple iteration, and as described [15], green/red indicate achievement/failure to achieve each threshold, with amber indicating that values are near to achieving the threshold

There are several potential reasons for the lack of substantial intervention effects on physical activity outcomes, including explanations related to the novel multidimensional approach that was employed in MIPACT for the first time. We recruited patients based on the presence of a specific score in a predefined physical activity dimension (PAL <2.0), and thus participants could still score relatively highly for other physical activity dimensions. To illustrate this point, the majority of participants (58%) scored highly (green) for at least one physical activity dimension at baseline and only 11 participants (5%) had universally low physical activity across all dimensions (Fig. 2). Thus, most participants would have received at least a partially positive message about some aspect of their physical activity behaviour from the online platform and during health trainer sessions. From a behaviour change standpoint, receiving positive feedback (even in just one dimension of physical activity) could reinforce the “appropriateness” of an individual’s existing behaviour and lessen the perceived need to change physical activity behaviour, especially in areas that participants do not think match their lifestyle and their preferences [12, 13]. Clinical practice might consider focusing on priority populations with uniformly low physical activity across all dimensions.

The lack of substantial intervention effects could also be explained by study inclusion based on absolute risk scores, which may be biased towards healthy older people. Disease-risk algorithms such as QRisk do not directly consider the impact of physical activity and estimated risk is heavily influenced by age [17]. To illustrate this point, a lean non-smoking 64-year old man with no family history or risk factors for cardiovascular disease would have been eligible for MIPACT with a 10-year QRISK score of 13.9%. Thus, inclusion was heavily influenced by age-related absolute risk score, which is a poor proxy for lifestyle-related health outcomes. Indeed, in MIPACT, participants had a slightly higher mean PAL (1.68) than the UK median (1.63) [39, 40]. Thus, Health Check programmes with a focus on prevention may need to avoid using absolute risk scores and consider targeting populations with higher relative risk.

### Strengths and limitations of this study

The current study has several important strengths. We used a precise device-based measure of physical activity and achieved exceptional 24-h, 7-day compliance (> 98%) across all assessment periods. We adopted an innovative approach to try and avoid short-term changes in physical activity during assessment periods by using sham activity monitors in the 1-month prior to follow-up [15]. Also, we adopted a pragmatic intention-to-treat (as randomised) analysis as well as an extended post-intervention follow-up assessment at 12 months. Participant retention in the trial was high (91%) and did not differ substantially between study groups. The study also had a number of limitations. Participants came from a single UK region, were well educated, and exhibited limited ethnic and socioeconomic diversity. As such, our findings may not be generalisable to other cohorts and settings, although representativeness is not a prerequisite for a valid evaluation of relative efficacy/ effectiveness in a randomised trial [41].

## Conclusions

In primary care patients at risk of cardiovascular disease and/or type II diabetes, a highly sophisticated digital monitoring system along with support from a health trainer (MIPACT) did not increase mean physical activity levels compared to usual care. MIPACT shows that physical activity is hard to change in patients recruited from primary care in the longer term, even with highly sophisticated digital technologies. The multidimensional individual-level characterisation revealed enormous heterogeneity in physical activity in primary care patients, and only a small proportion of patients had low physical activity across all dimensions. Practitioners may need to screen for inactivity across multiple outcomes/metrics rather than rely on disease-risk algorithms that are heavily influenced by age.

## Supplementary information

**Additional file 1.** CONSORT 2010 checklist of information to include when reporting a randomised trial*

**Additional file 2.** The TIDieR (Template for Intervention Description and Replication) Checklist*

## Data Availability

All the individual participant data collected during the trial (along with the data dictionary) will be available, after deidentification, immediately following publication with no end date. Data will be available to anyone and for any purpose. The study protocol has been published. Data are available indefinitely at: 10.15125/BATH-00713

## Abbreviations

MIPACT: Multidimensional Individualised Physical ACTivity
NHS: National Health Service
PAL: Physical Activity Level
MVPA: Moderate-to-Vigorous Physical Activity
METs: Metabolic Equivalents
MCID: Minimum Clinically Important Difference

## References

[1] Lee IM, Shiroma EJ, Lobelo F, Puska P, Blair SN, Katzmarzyk PT (2012). Effect of physical inactivity on major non-communicable diseases worldwide: an analysis of burden of disease and life expectancy. Lancet.

[2] Department of Health (2009). Putting prevention first - vascular checks: risk assessment and management.

[3] Sanchez A, Bully P, Martinez C, Grandes G (2015). Effectiveness of physical activity promotion interventions in primary care: a review of reviews. Prev Med.

[4] Phillips SM, Cadmus-Bertram L, Rosenberg D, Buman MP, Lynch BM (2018). Wearable technology and physical activity in chronic disease: opportunities and challenges. Am J Prev Med.

[5] Davies CA, Spence JC, Vandelanotte C, Caperchione CM, Mummery WK (2012). Meta-analysis of internet-delivered interventions to increase physical activity levels. Int J Behav Nutr Phys Act.

[6] Direito A, Carraca E, Rawstorn J, Whittaker R, Maddison R (2017). mHealth technologies to influence physical activity and sedentary behaviors: behavior change techniques, systematic review and meta-analysis of randomized controlled trials. Ann Behav Med.

[7] Michie S, Abraham C, Whittington C, McAteer J, Gupta S (2009). Effective techniques in healthy eating and physical activity interventions: a meta-regression. Health Psychol.

[8] Greaves CJ, Sheppard KE, Abraham C, Hardeman W, Roden M, Evans PH (2011). Systematic review of reviews of intervention components associated with increased effectiveness in dietary and physical activity interventions. BMC Public Health.

[9] O'Brien N, McDonald S, Araujo-Soares V, Lara J, Errington L, Godfrey A (2015). The features of interventions associated with long-term effectiveness of physical activity interventions in adults aged 55-70 years: a systematic review and meta-analysis. Health Psychol Rev.

[10] Thompson D, Peacock O, Western M, Batterham AM (2015). Multidimensional physical activity: an opportunity, not a problem. Exerc Sport Sci Rev.

[11] 2018 PAGAC (2018). Physical activity guidelines committee scientific report.

[12] Western MJ, Peacock OJ, Stathi A, Thompson D. The understanding and interpretation of innovative technology-enabled multidimensional physical activity feedback in patients at risk of future chronic disease. PLoS One. 2015. 10.1371/journal.pone.0126156. 10.1371/journal.pone.0126156 PMC4418766 25938455

[13] Western MJ, Thompson D, Peacock OJ, Stathi A. The impact of multidimensional physical activity feedback on healthcare practitioners and patients. BJGP Open. 2019. 10.3399/bjgpopen18X101628. 10.3399/bjgpopen18X101628 PMC6480860 31049409

[14] Thompson D, Batterham AM (2013). Towards integrated physical activity profiling. PLoS One.

[15] Peacock OJ, Western MJ, Batterham AM, Stathi A, Standage M, Tapp A (2015). Multidimensional individualised physical ACTivity (mi-PACT) - a technology-enabled intervention to promote physical activity in primary care: study protocol for a randomised controlled trial. Trials.

[16] Hippisley-Cox J, Coupland C, Robson J, Sheikh A, Brindle P (2009). Predicting risk of type 2 diabetes in England and Wales: prospective derivation and validation of QDScore. Br Med J.

[17] Hippisley-Cox J, Coupland C, Vinogradova Y, Robson J, Minhas R, Sheikh A (2008). Predicting cardiovascular risk in England and Wales: prospective derivation and validation of QRISK2. Br Med J.

[18] Dumville JC, Hahn S, Miles JNV, Torgerson DJ (2006). The use of unequal randomisation ratios in clinical trials: a review. Contemp Clin Trials.

[19] Treasure T, MacRae KD (1998). Minimisation: the platinum standard for trials? Randomisation doesn’t guarantee similarity of groups; minimisation does. Br Med J.

[20] Craig P, Dieppe P, Macintyre S, Michie S, Nazareth I, Petticrew M (2008). Developing and evaluating complex interventions: the new Medical Research Council guidance. Br Med J.

[21] van Middelaar T, Beishuizen CRL, Guillemont J, Barbera M, Richard E, Moll van Charante EP (2018). Engaging older people in an internet platform for cardiovascular risk self-management: a qualitative study among Dutch HATICE participants. BMJ Open.

[22] Michie S, Richardson M, Johnston M, Abraham C, Francis J, Hardeman W (2013). The behavior change technique taxonomy (v1) of 93 hierarchically clustered techniques: building an international consensus for the reporting of behavior change interventions. Ann Behav Med.

[23] Gillison FB, Rouse P, Standage M, Sebire SJ, Ryan RM (2019). A meta-analysis of techniques to promote motivation for health behaviour change from a self-determination theory perspective. Health Psychol Rev.

[24] Samdal GB, Eide GE, Barth T, Williams G, Meland E (2017). Effective behaviour change techniques for physical activity and healthy eating in overweight and obese adults; systematic review and meta-regression analyses. Int J Behav Nutr Phys Act.

[25] Ryan RM, Deci EL (2000). Self-determination theory and the facilitation of intrinsic motivation, social development, and well-being. Am Psychol.

[26] Ryan RM, Patrick H, Deci EL, Williams GC (2008). Facilitating health behaviour change and its maintenance: interventions based on self-determination theory. Eur Health Psychol.

[27] Standage M, Ryan RM, Roberts GC, Treasure DC (2012). Self-determination theory and exercise motivation: facilitating self-regulatory processes to support and maintain health and well-being. Advances in motivation in sport and exercise.

[28] Bellg AJ, Borrelli B, Resnick B, Hecht J, Minicucci DS, Ory M (2004). Enhancing treatment fidelity in health behavior change studies: best practices and recommendations from the NIH behavior change consortium. Health Psychol.

[29] Schofield WN (1985). Predicting basal metabolic rate, new standards and review of previous work. Clin Nutr.

[30] Johannsen DL, Calabro MA, Stewart J, Franke W, Rood JC, Welk GJ (2010). Accuracy of armband monitors for measuring daily energy expenditure in healthy adults. Med Sci Sports Exerc.

[31] Jakicic JM, Davis KK, Rogers RJ, King WC, Marcus MD, Helsel D (2016). Effect of wearable technology combined with a lifestyle intervention on long-term weight loss: the IDEA randomized clinical trial. JAMA.

[32] EuroQol (1996). A standardised instrument for use as a measure of health outcome.

[33] Frison L, Pocock SJ (1992). Repeated measures in clinical trials - analysis using mean summary statistics and its implications for design. Stat Med.

[34] Rothman KJ (1990). No adjustments are needed for multiple comparisons. Epidemiology.

[35] Bakrania K, Edwardson CL, Khunti K, Henson J, Stamatakis E, Hamer M (2017). Associations of objectively measured moderate-to-vigorous-intensity physical activity and sedentary time with all-cause mortality in a population of adults at high risk of type 2 diabetes mellitus. Prev Med Rep.

[36] Amrhein V, Trafinnow D, Greenland S (2019). Inferential statistics as descriptive statistics: there is no replication crisis if we don’t expect replication. Am Stat.

[37] van der Weegen S, Verwey R, Spreeuwenberg M, Tange H, van der Weijden T, de Witte L. It’s LiFe! Mobile and web-based monitoring and feedback tool embedded in primary care increases physical activity: a cluster randomized controlled trial. J Med Internet Res. 2015. 10.2196/jmir.4579. 10.2196/jmir.4579 PMC4529491 26209025

[38] Harris T, Kerry SM, Limb ES, Victor CR, Iliffe S, Ussher M, et al. Effect of a primary care walking intervention with and without nurse support on physical activity levels in 45-to 75-year-olds: the pedometer and consultation evaluation (PACE-UP) cluster randomised clinical trial. PLoS Med. 2017. 10.1371/journal.pmed.1002210. 10.1371/journal.pmed.1002210 PMC5207642 28045890

[39] Scientific Advisory Committee for Nutrition (2011). Dietary reference values for energy.

[40] Thompson D, Batterham AM, Peacock OJ, Western MJ, Booso R (2016). Feedback from physical activity monitors is not compatible with current recommendations: a recalibration study. Prev Med.

[41] Senn SJ (1991). Falsificationism and clinical trials. Stat Med.

